# *IBM WATSON Trauma Pathway Explorer©* as a Predictor for Sepsis after Polytrauma - Is Procalcitonin Useful for Identifying Septic Polytrauma Patients?

**DOI:** 10.26502/jsr.10020272

**Published:** 2022-12-19

**Authors:** Cédric Niggli, Philipp Vetter, Jan Hambrecht, Philipp Niggli, Jindřich Vomela, Richard Chaloupka, Hans-Christoph Pape, Ladislav Mica

**Affiliations:** 1 Department of Trauma Surgery, University Hospital Zurich, 8091 Zurich, Switzerland; 2 Department of Mathematics, ETH Zurich, 8092 Zurich, Switzerland; 3 Division of Medical Sciences in Sportsmedicine, Faculty of Sports Studies, Masaryks University, 62500 Brno, Czech Republic; 4 Department of Orthopedic Surgery, Masaryks University, 62500 Brno, Czech Republic

**Keywords:** Systemic Inflammatory Response Syndrome (SIRS), Polytrauma, Sepsis

## Abstract

IBM and the University Hospital Zurich have developed an online tool for predicting outcomes of a patient with polytrauma, the *IBM WATSON Trauma Pathway Explorer^®^*. The three predicted outcomes are Systemic Inflammatory Response Syndrome (SIRS) and sepsis within 21 days as well as early death within 72 hours since the admission of the patient. The validated *Trauma Pathway Explorer^®^* offers insights into the most common laboratory parameters, such as procalcitonin (PCT). Sepsis is one of the most important complications after polytrauma, which is why it is crucial to detect it early. This study aimed to examine the time-dependent relationship between PCT values and sepsis, based on the WATSON technology. A total of 3653 patients were included, and ongoing admissions are incorporated continuously. Patients were split into two groups (sepsis and non-sepsis), and the PCT value was assessed for 21 days (1, 2, 3, 4, 6, 8, 12, 24, 48 hours, and 3, 4, 5, 7, 10, 14 and 21 days). The Mann-Whitney U-Test was used to evaluate the difference between the two groups. Binary logistic regression was utilized to examine the dependency of prediction. The Closest Top-left Threshold Method provided time-specific thresholds at which the PCT level is predictive for sepsis. At p <0.05, the data were declared significant. R was used to conduct all statistical analyses. The Mann-Whitney U-test showed a significant difference in PCT values in sepsis and non-sepsis patients between 12 and 24 hours, including post-hoc analysis (p <0.05). Likewise, the p-value started to be significant between 12 and 24 hours in the binary logistic regression (p <0.05). The threshold value of PCT to predict sepsis at 24 hours is 0.7μg/l, and at 48 hours 0.5μg/l. The presented time course of PCT levels in polytrauma patients shows the PCT as a separate predictor for sepsis relatively early. Even later, during the 21-day observation period, time-dependent PCT values may be utilized as a benchmark for the early and preemptive detection of sepsis, which may reduce death from septic shock and other deadly infectious episodes.

## Introduction

In polytrauma patients, septic complications are the most common cause of death (>1 week) [1] Klicken oder tippen Sie hier, um Text einzugeben. Immunological exhaustion defines the Compensatory Anti-Inflammatory Response Syndrome (CARS), polytrauma patients are more likely to suffer infective sequelae such as sepsis [2,3]. Treating sepsis immediately, correctly, and successfully has been found to reduce multi-organ failure (MOF), and improve mortality and clinical outcomes [4]. In septic patients, each hour of delay in antibiotic therapy following the start of hypotension has been shown to increase mortality by 7.6% [4]. Therefore, biomarkers, patient demographics, and injury patterns have been the most common strategies for predicting sepsis in trauma patients thus far. However, few studies confirm their predictive value, and the results are still debatable [5]. A review by Ciriello et al. suggested that procalcitonin (PCT) might be used as an early predictor for sepsis in trauma patients [6]. PCT is an Acute Phase Protein and a precursor of the hormone calcitonin. In response to systemic bacterial infection or activation with endotoxin or proinflammatory cytokines, PCT levels can increase by 1000-fold [7]. PCT is typically elevated very early after trauma and quickly returns to baseline in uncomplicated recovery, often 24–48 hours after admission, due to the short half-life period (about 22 hours). After the first 48 hours, they remain elevated or increase in septic patients [8–13]. The damage control concept in the treatment of polytrauma patients takes into account avoiding inflammatory and infectious consequences. To forecast the outcome of critically injured patients, this study group worked with IBM to build the IBM WATSON Trauma Pathway Explorer©, an artificial intelligence (AI) tool [14,15]. This interactive tool includes clinical parameters that enable the prediction of different outcomes, such as early death within 72 hours since the admission and Systemic Inflammatory Response Syndrome (SIRS) as well as sepsis within 21 days since the admission of the patient. The tool also visually shows the timetable for frequently used laboratory variables in clinical practice, such as the PCT. Within hours of arrival, the PCT values between those who had sepsis and those who had not were different. At this point, it was suggested that improving the outcome would be possible by reducing PCT values through surgical or medical interventions relatively soon after the arrival of a critically injured patient. Undoubtedly, a thorough time analysis of PCT values was required. The objective of this study was to show these time-dependent PCT values in both the sepsis and non-sepsis groups.

## Methods

### Ethical Statement

The study was conducted in accordance with the guidelines for good clinical practice and the Helsinki guidelines. The TRIPOD Statement, a standard for multivariable prediction models, served as the foundation for this study [16]. The University Hospital Zurich’s ethics commission and the Zurich government approved the analysis of patient records upon the development of the database (Nr. StV: 1-2008), and they again approved it for the development of the WATSON Trauma Pathway Explorer© (BASEC: 2021-00391).

### WATSON Trauma Pathway Explorer©

The Pathway Explorer is an interactive visualization tool that organizes the data of polytrauma patients and presents it to the user in a consumable way. Different pathways through different events (coagulopathy, hemorrhagic shock class, surgical treatment strategy) lead to one of the three outcomes (SIRS, sepsis, early death). Furthermore, the tool also visually shows the timetable for frequently used laboratory variables in clinical practice, such as procalcitonin, C-reactive protein, or interleukin 6 [14,15].

### Inclusion/Exclusion Criteria

Eligibility criteria for the patients were age ≥16 years and Injury Severity Score (ISS) ≥16. A polytrauma is an ISS score equal to or greater than 16, which was also the definition used for this study [17]. Patients were admitted primarily to the trauma bay, and only those with complete datasets were considered. Patients referred from another hospital, as well as non-survivors on the scene, were excluded. During the 21-day observation period, the sample was divided into two groups: one without sepsis and one with sepsis. WATSON Trauma Pathway Explorer© was used on 3653 patients in total. The database was established on 01.08.1996, and data is still being collected for polytrauma patients.

### Sample Size and Power Analysis

The sample size was not calculated because the entire retrospective data set since 1996 with ongoing patient inclusion was used for the analysis. A power analysis is not necessary since we already calculated sensitivity for the Closest Top-left Threshold Method. We consider an independent power analysis to be pointless since it is not the values of the p-values that are crucial, but the development of the p-values over time.

### Definition of Sepsis

SIRS was defined as the presence of two or more of the following criteria: body temperature >38°C or <36°C, heart rate >90 bpm, respiratory rate >20 breaths/min, and white blood cell count >12’000/μl or <4’000/μl [18]. SIRS was measured for the first 30 days after admission or the duration of the patient’s hospitalization. Sepsis was defined as a SIRS score ≥2 with an additional infectious focus [18]. An infection had to be diagnosed either by strong clinical sepsis criteria (such as organ dysfunction, hypotension, hypoperfusion) or by microbiological detection. Sepsis had to occur at any time during the 21-day observation period. The sepsis definition used in this study was the older definition according to the ACCP/SCCM Consensus Conference Committee [18]. In recent years, an alternative definition for sepsis evolved (Sepsis-3 Criteria) [19]. However, all sepsis criteria used for the data collection in this cohort of the hospital were assessed according to the well-established older, more common definition. Some recent work has also stated the outperformance of the old sepsis definition [20].

### PCT Measurement

The PCT values measured were assigned to one of the following scheduled time points: 1, 2, 3, 4, 6, 8, 12, 24, 48 hours, and 3, 4, 5, 7, 10, 14, and 21 days after admission to the trauma bay of the University Hospital Zurich. In the range from admission to 48 hours, the PCT values were assigned to the scheduled time point which was nearest. After 48 hours, however, the measured time point could not be more than one day away from the scheduled time point. No imputation method was applied for the missing values. The PCT values were measured in the Department of Clinical Chemistry at the University Hospital Zurich. In 2007, a unit was changed and since then the values are given in ug/l (previously in ng/ml). At the same time, the cut-off value was adjusted according to the unit. At the end of December 2008, there was an internal method change from the TRACE technique (Brahms) to the ECLIA technique (Roche). The values of the new method were comparable to the values of the old method. The pathological threshold values remained unchanged.

### Statistical Analysis

The baseline characteristics of the patient’s sample were described through means with standard deviation (SD) for normally distributed numerical data, medians with interquartile ranges (IQR) for non-normally distributed numerical data as well as for ordinal data, and percentages for binary variables. The differences between these groups were assessed using the unpaired t-test for numerical variables and Mood’s median test for ordinal variables. A Q-Q-Plot was used to test the data for normality. As the data were not normally distributed and the variance was not equal, the Mann-Whitney U-Test was used to analyze the difference between the two groups. Binary logistic regression was performed to determine whether PCT has an impact on the development of sepsis or not. Hereby, ISS was held constantly in the logistic regression. A p-value <0.05 was considered significant. The Closest Top-left Threshold Method was used to determine PCT threshold values at various time points. This method calculates the threshold point that is closest to the top-left of the receiver operating characteristic (ROC) plot of each PCT time point.

An effect size such as the odds ratio was not used for this study for the following two reasons:

#### Difficulty of interpretation:

An odds ratio is defined as the ratio of the odds of event A in the presence of event B and the odds of event A in the absence of event B. This metric is to some extent interpretable (e.g., log OR above 1 or below 1). This is the case when the independent variable is also binary. If the independent variable is continuous, as in this study, it is even more complicated. It also makes no difference whether the log-odds ratio or only the odds ratio is used.

#### Lack of comparability:

It is not possible to compare the effect size (odds ratio) across models with different independent variables [21,22]. Therefore, the focus was on the metrics that can be interpretable and also comparable (like the p-value). Statistics were performed with R-4.0.2.

## Results

### Characteristics of the patient sample

Totally, 3653 patients were included. In both study groups (sepsis and non-sepsis), roughly 75% of participants were men (Table 1). Most trauma mechanisms were blunt. In the sepsis group, the ISS was significantly higher (30; IQR 25-41 vs. 25; IQR 17-34, p <0.001) (Table 1). Similar to the ISS, the sepsis group’s score for the APACHE II was significantly higher compared to that of the non-sepsis group (17; IQR 11-21 vs. 13; IQR 6-21, p <0.001) (Table 1). The mean hospital length of stay was 16.88 days (SD ±18.70). 3310 (out of 3653) patients were admitted to the ICU, with a mean ICU length of stay of 8.95 days (SD ±10.64). The numbers of missing values for each time point including the percentage of the total patient sample are as follows: 3’613 (98.9%) at admission, 3’653 (100%) at 1h, 3’648 (99.9%) at 2h, 3’641 (99.7%) at 3h, 3’638 (99.6%) at 4h, 3’636 (99.5%) at 6h, 3’635 (99.5%) at 8h, 3’556 (97.3%) at 12h, 3’247 (88.9%) at 24h, 3’050 (83.5%) at 48h, 2’962 (81.1%) at 3d, 3’009 (82.4%) at 4d, 3’100 (84.9%) at 5d, 3’114 (85.2%) at 7d, 3’210 (87.9%) at 10d, 3’332 (91.2%) at 14d, and 3’477 (94.4%) at 21d.

### Significant differences in PCT values in the sepsis and non-sepsis group

**Figure 1:**
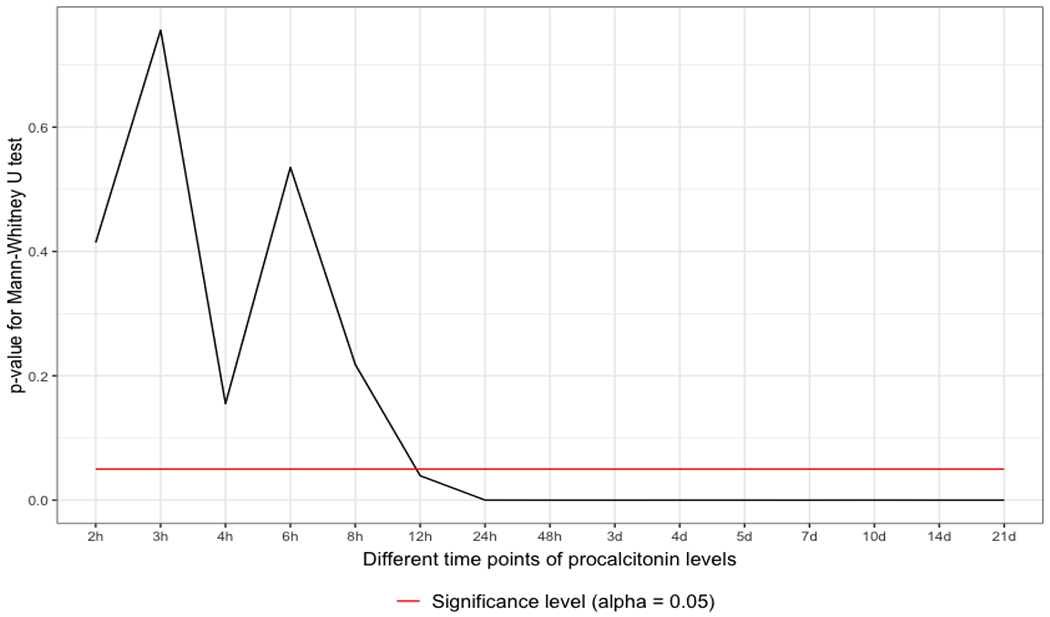
Mann-Whitney U-test between the sepsis and non-sepsis groups according to the points of time. As indicated, the differences start to be significant between 12 and 24 hours after admission (sepsis vs. non-sepsis).

The Q-Q plots have no normality shown. The data were tested with the Mann-Whitney U-test, which revealed significant differences between 12 and 24 hours after the admission of the polytrauma patient. The data remained significant 24 hours after admission and throughout the whole observational period (p <0.05) (Figure 1).

### Significant correlation of PCT values in the sepsis and non-sepsis group

**Figure 2:**
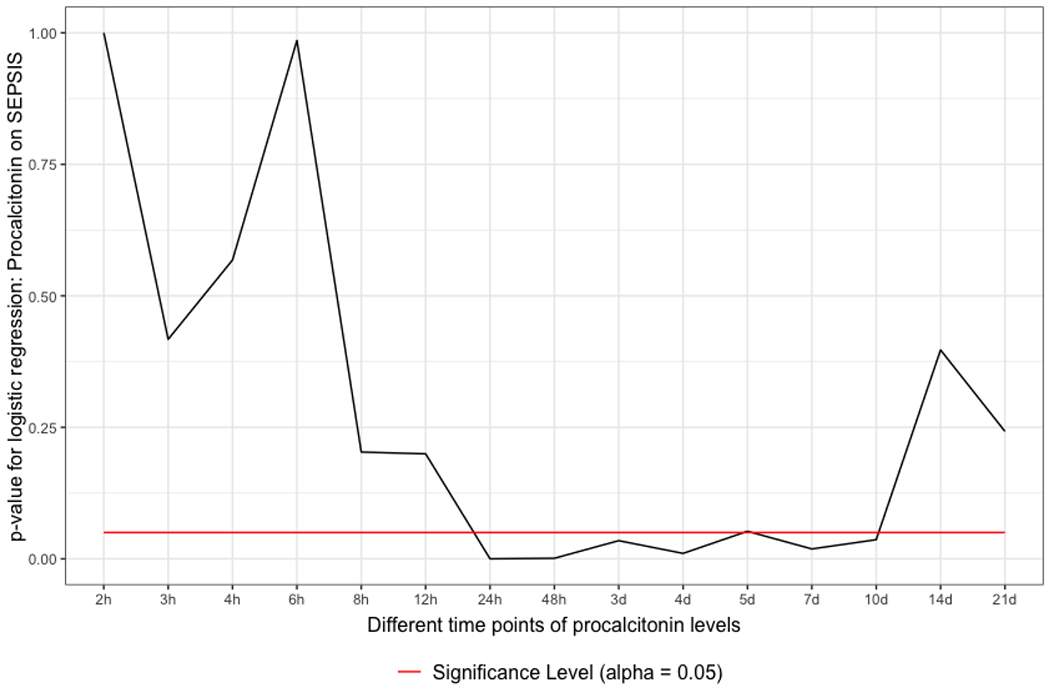
PCT is an independent predictor for sepsis. Binary logistic regression of the PCT values and the two groups (sepsis vs. non-sepsis). Also here, the values are significant between 12 and 24 hours.

As in the Mann-Whitney U-test, the binary logistic regression depicted a similar picture of significance. Between 12 and 24 hours following admission, the data started to be significant. However, they only persisted to be significant over 10 days and then started to have a p-value >0.05 (Figure 2).

### The predictive quality by the area under the ROC curve

No satisfactory values for the area under the ROC curve (AUROC) were revealed after the ROC analysis of the patient sample. The AUROC was always <0.800. In detail, AUROC is 0.62 after 12 hours and 0.64 after 24 hours.

### Threshold values as orientation points

**Figure 3:**
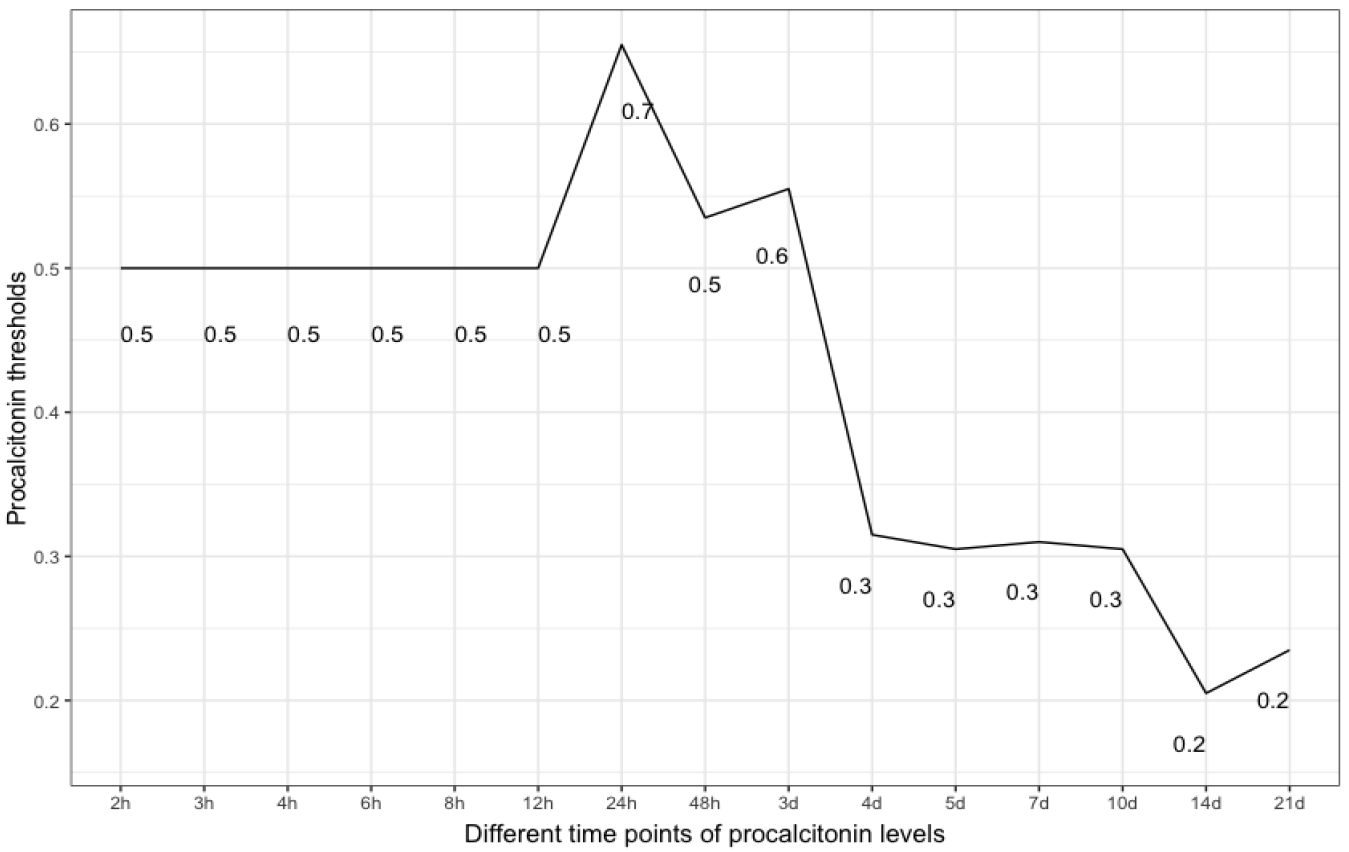
Closest Top-left Threshold Method of the patient sample. Shown are the threshold values of the PCT levels which are predictive for sepsis at a given time point (over the whole observational period of 21 days).

The patient sample was tested using the Closest Top-left Threshold Method. This diagram reflects the period of PCT values from 1 hour after admission to 21 days after admission (Figure 3). The result was a descending curve from 24 hours onwards, with a maximum peak of the PCT threshold value at 24 hours (0.7μg/l) (Figure 3).

## Discussion

The IBM WATSON Trauma Pathway Explorer© visualizes the PCT values in sepsis and non-sepsis cohorts at different points in time [14,15]. This led to the statistical, time-dependent analysis of PCT values to predict sepsis after polytrauma. The University Hospital Zurich does not have a systematic screening program for sepsis but follows international guidelines for sepsis treatment. During the time course of polytrauma management and definitive care, the PCT level might variate, and at some point in time, the diagnosis of sepsis might be set [23–25]. As first illustrated in figures 1 and 2, this fact can be applied between 12 and 24 hours following admission. In the absence of sepsis cases or the multiple etiology of PCT rise in a polytrauma patient, the poor predictive quality (AUROC) may be seen from a multivariate perspective. When considering the ICU treatment techniques and surgical operations that cause a rise in PCT values, the multivariate hypothesis appears to make sense. The time course of PCT values might aid a trauma surgeon in making decisions, keeping track of surgical success, and planning operations for second-look procedures. Early sepsis detection could reduce death from septic shock or other deadly infectious episodes. A preview of whether the PCT value is too high for the ISS circumstance is made feasible by treating the patient sample as ISS-corrected, which puts the PCT values on the same level. Healthy people have very low plasma concentrations of PCT (<0.1μg/l) [26]. A value of <0.2μg/l is a helpful reference point to exclude sepsis and systemic inflammation. Plasma values >0.5μg/l are considered abnormal and suggestive of sepsis and are used as a cut-off for the diagnosis [27]. As illustrated in figure 3, the PCT threshold values which are predictive for sepsis in this study were 0.5μg/l at 12 hours and 0.7μg/l at 24 hours. A systematic review by Ciriello et al. has demonstrated that PCT might be used as an early indicator of posttraumatic septic sequelae and that CRP was unable to specifically detect infective problems [6]. PCT has been shown to be effective in predicting sepsis early in trauma patients in five prospective cohort studies [7,8,10,12,28], two retrospective cohort studies [9,11], one prospective case-control study [29], and one retrospective case-control study [13]. PCT did not predict sepsis in two prospective trials, one cohort [30] and one case-control [31]. In several studies, PCT kinetics were fast, with levels peaking at 24–48 hours after trauma and rapidly declining in non-complicated patients [7,8,10,12,28]. Sepsis could be predicted by persistently high levels or secondary rises [7–13,29]. This study has shown similar results, with PCT values reaching their maximum after 24 hours. A previous study with the same polytrauma patient cohort at the University Hospital Zurich has shown that CRP is predictive for sepsis even earlier than PCT, 6-8 hours after admission to the trauma hospital [32] However, in many studies, CRP had no predictive ability for sepsis in trauma patients [8–12,28,30,33–35]. Further investigations have to be conducted to compare the predictive values of PCT with other biomarkers such as IL-10. According to a prospective case-control study by Giannoudis et al., patients with sepsis at the time of admission had significantly higher IL-10 levels [36]. This study has several limitations. First, hepatopathology and system senescence were not taken into account, nor was there any attempt to normalize weight or BMI. Second, the number of ventilated and intubated patients who entered the hospital cannot be determined. This may, however, be important since it could have an impact on respiratory infections that potentially cause sepsis. Third, the database was established on August 01, 1996, with ongoing patient data collection. It must be assumed that there have been fluctuations over the years regarding the admission of polytrauma patients and that not all patients have been consistently admitted to the register. Fourth, the two measurement methods for PCT throughout the whole time period might result in minor differences in PCT levels, although the pathological PCT threshold level remained unchanged. Fifth, no additional blood samples were taken from the patients as part of the study beyond the usual level, which is why precise adherence to the time schedule could not be guaranteed. Furthermore, there are a considerable number of missing values per time point, which may seem acceptable given the large patient sample. Moreover, no statement can be made about the long-term survival rate of the cohort. Finally, slight modifications to treatment guidelines during the past ten years may have affected PCT results following surgery.

## Conclusions

Rather than a single number, the time trend of PCT values seems to be the best predictor for septic periods after polytrauma. Further increases in PCT levels after the peak of 24-48 hours after trauma may indicate the onset of septic problems. The literature often promotes the superiority of PCT over CRP for predicting sepsis after trauma. However, this study has shown that this is not the case in the polytrauma cohort at the University Hospital Zurich. Further prospective studies must therefore show whether the findings are in concordance with the clinical reality of polytrauma patients.

## Figures and Tables

**Table 1:** Descriptive statistics of the patient sample. Shown are the PCT values for admission, 8, 12, 24, and 48 hours only.

	Patient sample N = 3653	Sepsis N = 547	No sepsis N = 3106	p-value
Age (mean, SD)	45.8±20.2	42.8±18.1	46.3±20.5	0.0002
Male	73.4%; N=2681	78.6%; N=430	72.4%; N=2251	-
Early death within 72h	19.3%; N=708	1.5%; N=8	22.5%; N=700	-
Blunt trauma	91.3%; N=3336	94.7%; N=518	90.7%; N=2818	-
Head injury	38.3%; N=1400	44.8%; N=245	37.2%; N=1155	-
BMI at admission (mean, SD)	25±4.4	25.9±4.4	24.8±4.3	<0.001
ISS (median, IQR)	25(17-34)	30(25-41)	25(17-34)	<0.001
NISS (median, IQR)	34(25-50)	41(33-50)	34(24-48)	<0.001
APACHE II at admission (median, IQR)	14(7-21)	17(11-21)	13(6-21)	<0.001
GCS at admission (median, IQR)	10(3-15)	3(3-14)	11(3-15)	<0.001
Temperature at admission (mean, SD)	35.5±1.7	35.4±1.7	35.6±1.7	0.131
Systolic blood pressure at admission (mean, SD)	130.7±27.6	128.5±27.7	131.2±27.5	0.0715
Quick at admission (median, IQR)	84(65-97)	80(61-92)	85(66-98)	0.1257
Hemoglobin at admission (mean, SD)	11.4±4	11±2.8	11.5±4.2	0.005
CRP at admission (mean, SD)	13.74±41.21	23.15±62.96	11.94±35.32	<0.001
pH at admission (mean, SD)	7.31±0.13	7.3 0±0.15	7.32±0.13	0.00632
Lactate at admission (mean, SD)	2.94±2.53	2.94±2.27	2.94±2.58	0.943
PCT at admission (median, IQR)	0.2(0.1-0.5)	0.3(0.09-0.7)	0.2(0.1-0.4)	1
PCT at 8 hours (median, IQR)	0(0-1)	1(0.5-1)	0(0-0.5)	0.2182
PCT at 12 hours (median, IQR)	0(0-1)	1(0-1)	0(0-1)	0.03925
PCT at 24 hours (median, IQR)	0.43(0.2-1.17)	0.82(0.29-4.475)	0.38(0.195-0.995)	<0.001
PCT at 48 hours (median, IQR)	0.47(0.2-1.39)	0.855(0.28-2.87)	00.4(0.2-1.07).	<0.001

## Data Availability

All data are available upon reasonable request. None of these data are available to a broad public. All data are stored in the clinical information system (KISIM) of the University Hospital Zurich.
